# Dissecting the heterogeneity of posttraumatic stress disorder: differences in polygenic risk, stress exposures, and course of PTSD subtypes

**DOI:** 10.1017/S0033291721000428

**Published:** 2022-11

**Authors:** Laura Campbell-Sills, Xiaoying Sun, Karmel W. Choi, Feng He, Robert J. Ursano, Ronald C. Kessler, Daniel F. Levey, Jordan W. Smoller, Joel Gelernter, Sonia Jain, Murray B. Stein

**Affiliations:** 1 Department of Psychiatry, University of California San Diego, La Jolla, CA, USA; 2 Biostatistics Research Center, Herbert Wertheim School of Public Health and Human Longevity Science, University of California San Diego, La Jolla, CA, USA; 3 Department of Psychiatry, Massachusetts General Hospital, Boston, MA, USA; 4 Department of Epidemiology, Harvard T.H. Chan School of Public Health, Boston, MA, USA; 5 Psychiatric and Neurodevelopmental Genetics Unit, Center for Genomic Medicine, Massachusetts General Hospital, Boston, MA, USA; 6 Stanley Center for Psychiatric Research, Broad Institute, Boston, MA, USA; 7 Center for the Study of Traumatic Stress, Department of Psychiatry, Uniformed Services University of the Health Sciences, Bethesda, MD, USA; 8 Department of Health Care Policy, Harvard Medical School, Boston, MA, USA; 9 Department of Psychiatry, Genetics, and Neuroscience, Yale University School of Medicine, New Haven, CT, USA; 10 Veterans Affairs Connecticut Healthcare System, West Haven, CT, USA; 11 Herbert Wertheim School of Public Health and Human Longevity Science, University of California San Diego, La Jolla, CA, USA; 12 Veterans Affairs San Diego Healthcare System, San Diego, CA, USA

**Keywords:** Posttraumatic stress disorder, typology, latent class analysis, polygenic risk scores, military personnel

## Abstract

**Background:**

Definition of disorder subtypes may facilitate precision treatment for posttraumatic stress disorder (PTSD). We aimed to identify PTSD subtypes and evaluate their associations with genetic risk factors, types of stress exposures, comorbidity, and course of PTSD.

**Methods:**

Data came from a prospective study of three U.S. Army Brigade Combat Teams that deployed to Afghanistan in 2012. Soldiers with probable PTSD (PTSD Checklist for Diagnostic and Statistical Manual of Mental Disorders-Fifth Edition ≥31) at three months postdeployment comprised the sample (*N* = 423) for latent profile analysis using Gaussian mixture modeling and PTSD symptom ratings as indicators. PTSD profiles were compared on polygenic risk scores (derived from external genomewide association study summary statistics), experiences during deployment, comorbidity at three months postdeployment, and persistence of PTSD at nine months postdeployment.

**Results:**

Latent profile analysis revealed profiles characterized by prominent intrusions, avoidance, and hyperarousal (threat-reactivity profile; *n* = 129), anhedonia and negative affect (dysphoric profile; *n* = 195), and high levels of all PTSD symptoms (high-symptom profile; *n* = 99). The threat-reactivity profile had the most combat exposure and the least comorbidity. The dysphoric profile had the highest polygenic risk for major depression, and more personal life stress and co-occurring major depression than the threat-reactivity profile. The high-symptom profile had the highest rates of concurrent mental disorders and persistence of PTSD.

**Conclusions:**

Genetic and trauma-related factors likely contribute to PTSD heterogeneity, which can be parsed into subtypes that differ in symptom expression, comorbidity, and course. Future studies should evaluate whether PTSD typology modifies treatment response and should clarify distinctions between the dysphoric profile and depressive disorders.

## Introduction

Investigation is needed to guide precision treatment for posttraumatic stress disorder (PTSD), a disabling condition that affects 6–8% of Americans (Goldstein *et al*., 2016; Kilpatrick *et al*., 2013). One foundation of precision treatment is the characterization of disorder subtypes that differ in terms of symptom expression, etiology, or other factors that may modify treatment response (Stein & Smoller, 2018). Studies of PTSD heterogeneity in general population (Campbell, Trachik, Goldberg, & Simpson, 2020; Pietrzak *et al*., 2014), first responder (Horn *et al*., 2016), and veteran (Byrne, Harpaz-Rotem, Tsai, Southwick, & Pietrzak, 2019) samples offer evidence of subtypes defined by prominent intrusions, avoidance, and hyperarousal (‘threat-reactivity’ class), negative affect and adhedonia (‘dysphoric’ class), and high levels of all PTSD symptoms (‘high-symptom’ class); raising the possibility that distinct PTSD variants could be targeted with tailored therapeutics.

The aim of the current study was to identify PTSD subtypes among recently deployed Army soldiers and to evaluate cross-profile differences in genetic risk factors, stress exposures, comorbidity, and course of PTSD. The study has the potential to advance understanding of PTSD subtypes in three ways. First, although researchers have recently looked to polygenic risk scores (PRSs) to understand the divergence of long-term outcomes of trauma exposure (Waszczuk *et al*., 2020), the value of PRSs for explaining PTSD heterogeneity/typology has not been evaluated. Evidence of distinct genetic risk profiles would offer both etiological insights and validation of PTSD typology from a biological standpoint. Second, prior studies of PTSD typology have used cross-sectional data, precluding inferences about causal links between specific traumatic experiences and PTSD subtypes, and evaluation of whether typology affects the course of PTSD. Finally, PTSD subtypes have not been examined in recently deployed service members, a population whose trauma exposure includes unique complications that could have implications for typology (e.g. moral injury, repetitive exposure, continued stress with postdeployment re-integration).

We hypothesized that a latent profile analysis (LPA) of PTSD symptom data from recently deployed soldiers would reveal PTSD subtypes resembling previously described threat-reactivity, dysphoric, and high-symptom classes. Extrapolating from prior evidence (Horn *et al*., 2016; Pietrzak *et al*., 2014), we predicted that high combat exposure would be associated with the threat-reactivity and high-symptom profiles and that traumatic loss and personal life stress during deployment would be associated with the dysphoric profile. The high-symptom profile was expected to display the most psychiatric comorbidity.

We also made tentative predictions regarding cross-profile differences in genetic risk. First, we hypothesized that the dysphoric profile would exhibit a higher polygenic risk for major depressive disorder (MDD) than the threat-reactivity profile, given that symptoms of mood disturbance are more prominent in the former subtype. Second, we predicted the high-symptom profile would display higher polygenic risk for ADHD than the other profiles, as difficulties in concentrating and externalizing behaviors appear most pronounced in the high-symptom subtype (Byrne *et al*., 2019). Finally, because a link has been proposed between polygenic risk for schizophrenia and re-experiencing (Gelernter *et al*., 2019), we predicted that the dysphoric profile would display lower polygenic risk for schizophrenia than the other profiles, given that the dysphoric subtype is characterized by relatively low levels of flashbacks and other intrusions (Byrne *et al*., 2019; Horn *et al*., 2016; Pietrzak *et al*., 2014).

## Methods

### Participants/overview

The Pre/Postdeployment Study (PPDS) of the Army Study to Assess Risk and Resilience in Servicemembers (Army STARRS; Kessler *et al*., 2013a; Ursano *et al*., 2014) is a prospective, multi-wave panel survey of three U.S. Army Brigade Combat Teams (BCTs) that deployed to Afghanistan in 2012 for an average of ten months. Baseline (T0) evaluation occurred one-to-two months before deployment. Follow-ups were conducted approximately one month (T1), three months (T2), and nine months (T3) postdeployment. Surveys were conducted at the BCTs’ home posts, except the T3 survey was conducted via web or telephone. Written informed consent was obtained for survey participation, linkage of survey responses to Army/Department of Defense administrative records, and having blood drawn for a genetics study. Procedures were approved by the Human Subjects Committees of the collaborating institutions.

Participants with probable *Diagnostic and Statistical Manual of Mental Disorders* (5th edition; DSM-5; American Psychiatric Association, 2013) PTSD at T2 were eligible for inclusion in this study. Probable DSM-5 PTSD was defined as a score ≥31 on survey items corresponding to the PTSD Checklist for DSM-5 (PCL-5; Blevins, Weathers, Davis, Witte, and Domino, 2015). Total PCL-5 scores of 31–33 can be used to identify probable DSM-5 PTSD (Bovin *et al*., 2016); a more liberal cut-score of 31 was used here because the possibility of including subthreshold cases was not problematic from a theoretical standpoint. Additionally, the use of this cut-score is consistent with the methods of a previous investigation of DSM-5 PTSD typology (Byrne *et al*., 2019). Among 6310 respondents with PTSD symptom data at T2, 423 were determined to have probable DSM-5 PTSD and were included in the LPA. PPDS respondents with probable DSM-5 PTSD at T3 but not at T2 (*n* = 317 of those who participated at all waves) were not included in the study.

To evaluate the effect of typology on the course of PTSD, an analysis of the persistence of PTSD from T2 to T3 was conducted in a subsample (*n* = 314) that provided PTSD symptom data at T3. Soldiers with *v*. without PTSD data at T3 did not differ on PTSD profile membership, nor did they differ on the majority of socio-demographic, Army service, deployment stress, and mental health variables examined in this study. However, those with PTSD symptom data at T3 were older on average (mean age = 26.6 *v*. 23.7; *p* *<* 0.001), more likely to be married (58.7% *v*. 35.8%; *p* *<* 0.001), and more likely to have previously deployed (57.3% *v*. 37.0%; *p* *<* 0.001), relative to those who did not have PTSD symptom data at T3.

PRSs were derived based on large-scale genome-wide association studies (GWASs) that have been conducted only among individuals of European ancestry, which do not predict well into samples of other ancestries (Martin *et al*., 2017). Thus, PRS analyses were conducted using a subsample of 250 soldiers of genetically determined European ancestry (Stein *et al*., 2016). A sensitivity analysis was performed to evaluate whether the characteristics of the PTSD profiles in the PRS subsample were comparable to those observed in the full study sample.

### Measures

*Posttraumatic stress disorder*. Assessment of past-30-day PTSD at T2 was based on items from the PTSD Checklist-Civilian Version (PCL-C; Weathers, Litz, Herman, Huska, and Keane, 1993) and the PCL-5 (Blevins *et al*. 2015). Respondents rated how much they were bothered by each symptom on a scale from “not at all” to “extremely” bothered (coded 0-4). Ratings of 20 survey items corresponding to PCL-5 items were indicators for the LPA. In addition, a PCL-5 total score (theoretical range = 0–80) and subscales quantifying intrusions, avoidance, negative affect, anhedonia, externalizing behavior, anxious arousal, and dysphoric arousal were derived (Armour *et al*., 2015). Raw PCL-5 subscale scores are not readily comparable due to their differing numbers of items. For ease of comparison, PCL-5 subscale scores were standardized (*M* = 0, s.d. = 1).

*Pre-deployment characteristics*. The T0 variables considered in relation to PTSD profile at T2 included age, sex, race, ethnicity, education, marital status, prior deployments (0, 1, ≥2), and lifetime history of PTSD. The lifetime PTSD diagnosis at T0 was based on ratings of PCL-C items (i.e. DSM-IV PTSD criteria). This and other Army STARRS survey-based diagnoses have demonstrated satisfactory concordance with diagnoses from independent structured clinical interviews (Kessler *et al*., 2013b). Parental history of depression was examined to complement the analysis of MDD PRS, and assessed with an item inquiring if the soldier's biological mother or father ever had “times lasting two weeks or longer when they were so depressed they couldn't concentrate, felt worthless, or felt their life was not worth living.”

*Deployment experiences.* Previous reports describe T1 measures of combat exposure (Campbell-Sills *et al*., 2020) and personal life stress during the index deployment (Campbell-Sills *et al*., 2018). The combat exposure scale (theoretical range = 0–9) captured experiences such as going on combat patrols and firing rounds/taking enemy fire. The personal life stress scale (theoretical range = 0–20) quantified stress during deployment due to finances, romantic relationships, legal problems, family relationships, and problems experienced by loved ones.

*Comorbidity at T2 and persistence of PTSD at T3*. The T2 survey included items adapted from the Composite International Diagnostic Interview Screening Scales (Kessler & Ustun, 2004) that were used to derive 30-day mental disorder diagnoses (Kessler *et al*., 2013b). Past-30-day suicidal ideation was evaluated using an expanded self-report version of the Columbia Suicidal Severity Rating Scale (Posner *et al*., 2011). Past-30-day MDD, generalized anxiety disorder (GAD), substance use disorder (SUD), and suicidal ideation at T2 were considered in relation to PTSD profile.

We also examined the persistence of PTSD at T3. The presence of probable PTSD at T3 was defined as a total PCL-5 score of ≥31, whereas the absence of probable PTSD was defined as a total PCL-5 score of *<*31 or, for phone participants, being skipped out of the PTSD section of the interview after failing to rate at least two of seven initial PTSD items as ‘a little bit’ or more bothersome.

*Polygenic risk scores*. Genotyping (on Illumina OmniExpress or PsychChip arrays), imputation, and genomic quality control procedures are detailed elsewhere (Stein *et al*., 2016). PRS was generated using the gtx R package incorporated in PRSice v2.0 software (Choi & O'Reilly, 2019). PRS was calculated as the sum of risk alleles carried at each variant weighted by each variant's estimated effect size, based on publicly available GWAS summary statistics from the Psychiatric Genomics Consortium for ADHD (Demontis *et al*., 2019) and schizophrenia (Schizophrenia Working Group of the Psychiatric Genomics Consortium, 2014) and from a recent Million Veteran Program meta-analysis for MDD (Levey *et al*., in press). To reduce inclusion of highly correlated variants, *p*-value-informed clumping was conducted with a linkage disequilibrium (LD) cutoff of *R*^2^ = 0.05 within a 500-kb window, excluding the major histocompatibility complex region of the genome and using European samples of the 1000 Genomes Project as the LD reference panel. PRS for MDD included variants whose effects met a *p* value threshold (*p_t_*) = .0001, as the PRS derived from this *p_t_* was most predictive of lifetime MDD in an independent sample (Army STARRS New Soldier Study). The PRSs for ADHD and schizophrenia were *a priori* chosen to use the same *p_t_*, although PRSs from a range of *p* value thresholds were subsequently examined in sensitivity analyses. PRSs were standardized within this sample of soldiers with probable PTSD (*M* = 0, s.d. = 1). Positive standardized scores indicate that a soldier's PRS (or a subgroup's mean PRS) is higher than the average PRS for the entire sample, while negative standardized scores indicate that a soldier's PRS (or a subgroup's mean PRS) is lower than the average PRS for the entire sample.

### Data analysis

Twenty PTSD symptom ratings corresponding to PCL-5 items were indicators for LPA using Gaussian mixture modeling with equal variances across profiles and covariances fixed to 0. Analyses were conducted in R version 3.6.1 (R Core Team, 2016) with the tidyLPA and mclust packages (Rosenberg *et al*., 2019). Models with one to five profiles were compared on overall fit, interpretability, and parsimony. Due to concerns about the generalizability of small latent classes, and consistent with other investigations of PTSD typology (Byrne *et al*., 2019; Horn *et al*., 2016), we also examined pofile sizes and considered it favorable for each profile in a model to represent at least 20% of the sample. Fit statistics considered were the log-likelihood, Akaike information criterion (AIC), Bayes information criterion (BIC), sample size-adjusted BIC, entropy, and bootstrapped likelihood ratio test (BLRT). Following model selection, we compared profile characteristics using Fisher's exact test and analysis of variance for categorical and continuous variables, respectively. When omnibus tests were significant, pairwise comparisons were conducted using *t* tests. Associations of PTSD profile with PRS were evaluated in linear regression models, adjusting for 10 ancestral principal components. Mean standardized PRS for each profile (with 95% confidence intervals) were estimated based on these models. Two-tailed *p* *<* 0.05 was considered statistically significant, except for pairwise comparisons where a Bonferroni correction was applied and two-tailed *p* *<* 0.017 was considered statistically significant.

## Results

The sample for LPA was predominantly male (94.1%), White (71.6%; 7.1% Black, 5.7% Asian, and 15.6% Others), and non-Hispanic (83.6%). Mean age was 25.9 years (s.d. = 6.0). More than one-third (35.2%) of the sample reported lifetime PTSD prior to the index deployment.

Results of LPA are shown in Table 1. The AIC, BIC, and sample size-adjusted BIC did not reach a minimum, and the BLRT was significant for each successive model. Given that a solid statistical rationale for model selection was lacking (i.e. fit simply improved with increasing model complexity), we prioritized our other model selection criteria. The three-profile model was readily interpretable, with profiles resembling classes described in prior studies and each profile representing at least 20% of the sample. Based on these factors, the three-profile model was considered a viable candidate for the final model. Increasing model complexity by extracting another profile yielded a four-profile model that included two groups differentiated solely on the basis of overall symptom severity (i.e. one profile with mean ratings of ~2 for most PCL-5 items and another with mean ratings of ~3 for most PCL-5 items). This difference of degree was deemed less likely to have important clinical or etiological implications than differences in *patterns* of symptom endorsement. The four-profile model also included a profile representing *<*20% of the sample. These limitations (differences of degree rather than kind; small class sizes) were accentuated in the five-profile model. We concluded that the additional profiles represented in the four- and five-profile models were not compelling enough from a theoretical or clinical standpoint to override considerations of parsimony, evidence from prior investigations, and concerns about the generalizability of small classes; thus, we accepted the three-profile solution.

**Table 1.** Results of a latent profile analysis of PCL-5 symptom ratings (*N* = 423)

Number of profiles	Log likelihood	AIC	BIC	SABIC	Entropy	BLRT	*p*
1	−13 273	26 788	26 788	26 661	1	–	–
2	−12 656	25 434	25 681	25 487	0.93	1235	*<*0.01
3	−12 366	24 897	25 229	24 968	0.88	579	*<*0.01
4	−12 249	24 703	25 120	24 793	0.86	235	*<*0.01
5	−12 139	24 526	25 028	24 634	0.88	220	*<*0.01

*Note*. PCL-5 = Posttraumatic Stress Disorder Checklist for DSM-5; AIC = Akaike information criterion; BIC = Bayes information criterion; SABIC = sample size-adjusted BIC; BLRT = bootstrapped likelihood ratio test.

**Fig. 1.**
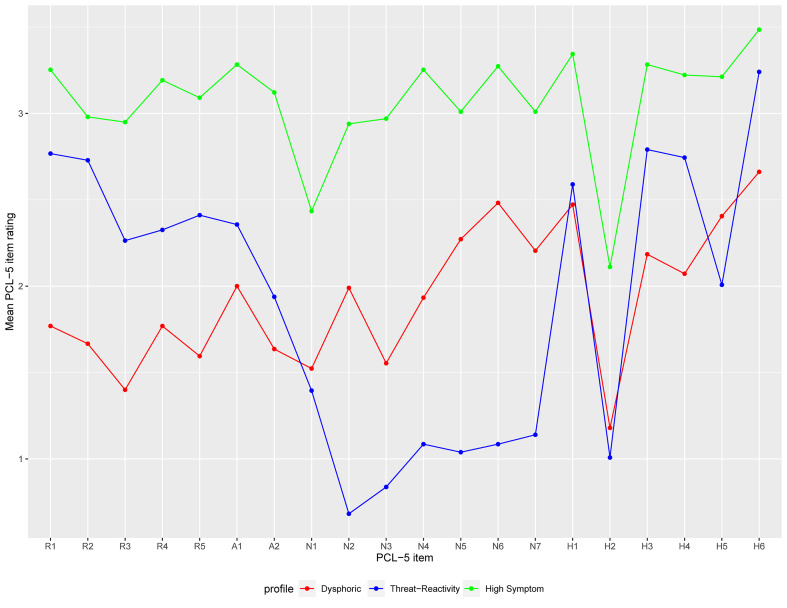
Mean PCL-5 item ratings for members of three latent PTSD profiles. Item ratings are coded 0 (‘not at all’ bothered by the symptom) to 4 (‘extremely’ bothered by the symptom). PCL-5 = PTSD Checklist for DSM-5; PTSD = posttraumatic stress disorder; R1 = intrusive memories; R2 = repeated dreams; R3 = flashbacks; R4 = upset by reminders; R5 = physical reactions to reminders; A1 = avoidance of internal cues; A2 = avoidance of external reminders; N1 = trouble remembering; N2 = strong negative beliefs; N3 = blaming self or others; N4 = strong negative emotions; N5 = loss of interest; N6 = feeling distant from others; N7 = trouble experiencing positive emotions; H1 = irritable behavior; H2 = excessive risk-taking; H3 = hypervigilance; H4 = easily startled; H5 = difficulty concentrating; H6 = sleep problems.

Figure 1 shows mean PCL-5 item ratings for each profile and online Supplementary Fig. S1 shows the proportion of each profile that endorsed each PTSD symptom at a moderate or worse level. Prominent symptoms among members of the first profile (*n* = 195; 46.1%) included feeling distant from other people, loss of interest, trouble experiencing positive emotions, irritable behavior, concentration problems, and sleep problems. Members of the first profile reported low levels of intrusion symptoms. Members of the second profile (*n* = 129; 30.5%) had high levels of intrusions, avoidance, and hyperarousal; and low levels of negative affect and anhedonia. Members of the third profile (*n* = 99; 23.4%) exhibited high levels of all PTSD symptoms. Following precedent (Byrne *et al*., 2019; Campbell *et al*., 2020; Horn *et al*., 2016), we labeled these the dysphoric, threat-reactivity, and high-symptom profiles, respectively.

**Fig. 2.**
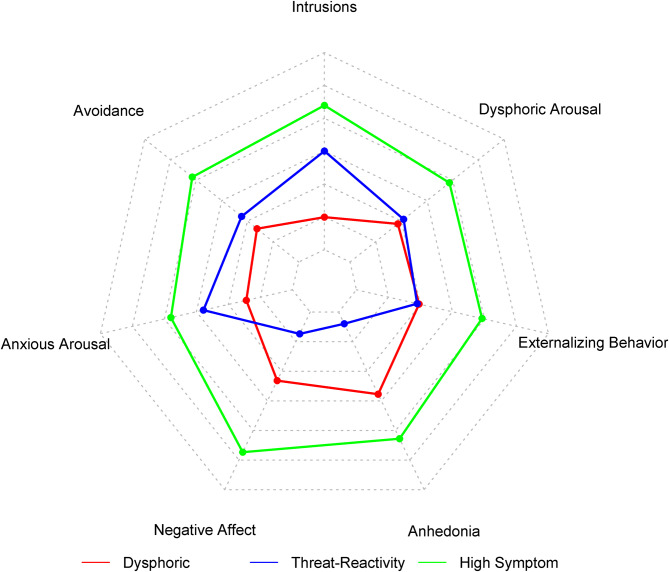
Radar plot displaying mean standardized PCL-5 subscale scores by latent PTSD profile. The inner axis has a minimum of −1.2 and a maximum of 1.8, with an interval of 0.5 between lines. PCL-5 = PTSD checklist for DSM-5; PTSD, posttraumatic stress disorder.

The high-symptom profile had a markedly higher mean PCL-5 total score (*M* = 61.4, s.d. = 8.2) than the dysphoric (*M* = 38.8, s.d. = 5.4; *p* *<* 0.001) and threat-reactivity (*M* = 38.4, s.d. = 6.3; *p* *<* 0.001) profiles. Furthermore, the high-symptom profile scored higher than the other profiles on all seven PTSD symptom clusters (*p* values < 0.001; Fig. 2). The dysphoric profile scored higher than the threat-reactivity profile on negative affect and anhedonia (*p* values < 0.001), whereas the threat-reactivity profile scored higher than the dysphoric profile on intrusions (*p* *<* 0.001), avoidance (*p* = 0.005), and anxious arousal (*p* *<* 0.001; Fig. 2).

Sociodemographic characteristics and deployment history did not differ significantly by profile (online Supplementary Table S1). Pre-deployment lifetime PTSD was related to profile membership (*p* = 0.004), with the high-symptom profile displaying a higher rate of pre-deployment PTSD (46.5%) than the threat-reactivity profile (25.6%; *p* = 0.001), and the dysphoric profile exhibiting an intermediate rate (35.9%) that did not differ significantly from the other profiles.

Combat exposure during the index deployment varied by profile, with members of the threat-reactivity profile reporting more overall combat exposure than members of the other profiles (Table 2). In terms of specific experiences (online Supplementary Table S2), members of the threat-reactivity profile had more exposure to combat patrols, firing rounds/being fired on, close calls, and seeing dead/wounded people than members of the dysphoric profile; and were more likely to report having five or more unit members seriously wounded or killed than members of the high-symptom profile. Personal life stress also varied by profile, with members of the dysphoric profile reporting significantly more personal life stress during deployment than members of the threat-reactivity profile (Table 2). The high-symptom profile had substantially more comorbidity than the other profiles; and a greater proportion of the high-symptom profile had probable PTSD at T3, relative to the threat-reactivity and dysphoric profiles (Table 2).

**Table 2.** Deployment stress and postdeployment clinical characteristics of three latent PTSD profiles

	Dysphoric (*n* = 195)	Threat reactivity (*n* = 129)	High symptom (*n* = 99)	*p*	Pairwise results
Combat exposure at T1 (mean, s.d.)^1^	3.5 (2.2)	4.7 (2.1)	3.8 (2.2)	*<*0.001	**D** *v.* **T *p* *<* 0.001***;* D *v.* H *p* = 0.21*;* **T *v.* H *p* =** 0**.006**
Personal life stress at T1 (median, IQR)^2^	6.0 (3.0–9.0)	3.5 (1.0–7.0)	5.0 (2.3–9.0)	0.008	**D *v.* T** *p* **=** 0**.002***;* D *v.* H *p* = 0.70*;* T *v.* H *p* = 0.04
Comorbid MDD at T2				*<*0.001	**D *v.* T *p* *<* 0.001**; **D *v.* H *p* *<* 0.001**; **T *v.* H *p* *<* 0.001**
No	82 (42.1%)	94 (72.9%)	16 (16.2%)		
Yes	113 (57.9%)	35 (27.1%)	83 (83.8%)		
Comorbid GAD at T2				*<*0.001	**D *v.* T *p* =** 0**.01**; **D *v.* H *p* *<* 0.001**; **T *v.* H *p* *<* 0.001**
No	117 (60.0%)	95 (73.6%)	19 (19.2%)		
Yes	78 (40.0%)	34 (26.4%)	80 (80.8%)		
Comorbid SUD at T2				0.003	D *v*. T *p* = 0.22; D *v*. H *p* = 0.02; **T *v*. H *p* =** 0**.001**
No	158 (81.0%)	112 (86.8%)	68 (68.7%)		
Yes	37 (19.0%)	17 (13.2%)	31 (31.3%)		
Comorbid suicidal ideation at T2				*<*0.001	**D *v*. T *p* *<* 0.001**; D *v*. H *p* = 0.10*;* **T *v*. H *p* *<* 0.001**
No	157 (80.5%)	125 (96.9%)	71 (71.7%)		
Yes	38 (19.5%)	4 (3.1%)	28 (28.3%)		
Probable PTSD at T3^3^				0.001	D *v*. T *p* = 0.36; **D *v*. H *p* *<* 0.001**; **T *v*. H *p* =** 0**.009**
Negative	77 (55.8%)	49 (49.5%)	23 (29.9%)		
Positive	61 (44.2%)	50 (50.5%)	54 (70.1%)		

*Note*. Unless otherwise indicated, values are No. (%). Bold type denotes pairwise comparisons that were statistically significant after Bonferroni correction (*p* *<* 0.017). PTSD = posttraumatic stress disorder; MDD = major depressive disorder; GAD = generalized anxiety disorder; SUD = substance use disorder; D = dysphoric profile; T = threat-reactivity profile; H = high-symptom profile.

1 The combat exposure score was missing for 35 participants.

2 The life stress score was missing for 25 participants. The distribution of scores was significantly skewed; thus, median (IQR) are reported. A Kruskal–Wallis test was used to evaluate between-groups differences, with subsequent Wilcoxon Rank Sum tests for pairwise comparisons.

3 The analysis of PTSD persistence was based on a subsample of 314 respondents who had PCL-5 data at nine months postdeployment. Missing data were due to non-participation in the T3 survey (*n* = 64) or missing PCL-5 score (*n* = 45).

As explained in the ‘Methods’ section, the analysis of cross-profile differences in polygenic risk for MDD, ADHD, and schizophrenia was conducted in a subsample of genetically determined European ancestry (*n* = 250). Results of sensitivity analyses indicated that the characteristics of the three PTSD profiles in soldiers of European ancestry were consistent with the characteristics of the PTSD profiles in the full study sample (see online Supplementary Table S3 for full results of the sensitivity analysis). In most cases, both the pattern and statistical significance of the results of cross-profile comparisons remained intact in the PRS subsample. The associations of PTSD profile with personal life stress during deployment and with some specific combat experiences were non-significant in the subsample; however, in those cases the pattern of results was analogous to that described above for the full sample.

**Fig. 3.**
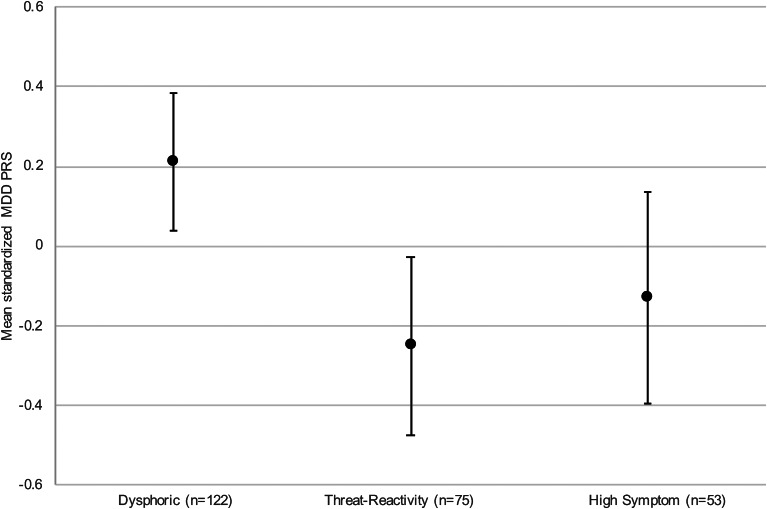
Estimated mean standardized MDD polygenic risk scores by PTSD profile. Error bars display 95% confidence intervals for the means. Estimates are from a linear regression model that adjusted for 10 ancestral principal components. MDD, major depressive disorder; PTSD, posttraumatic stress disorder.

Results of the regression adjusting for ancestral variables supported the hypothesis that the dysphoric profile would display higher polygenic risk for MDD than the threat-reactivity profile (dysphoric *v.* threat reactivity: *b* = 0.46, s.e. = 0.14, *t* = 3.21, *p* = 0.002), and further suggested that the dysphoric profile had elevated polygenic risk for MDD relative to the high-symptom profile (dysphoric *v.* high symptom: *b* = 0.34, s.e. = 0.16, *t* = 2.11, *p* = 0.04). Figure 3 shows the estimated mean standardized MDD PRS for each of the PTSD profiles, based on the regression model. In the full sample, profile membership was also associated with parental history of depression (*p* = 0.03), with a greater proportion of the dysphoric profile (31.8%) reporting parental depression than the threat-reactivity profile (18.6%; *p* = 0.01), and the high-symptom profile reporting an intermediate rate (27.3%) that did not differ significantly from the other profiles. In the PRS subsample, the pattern of results for parent history of depression was similar, but the between-groups difference was not statistically significant (online Supplementary Table S3).

The hypothesis that the high-symptom profile would have elevated ADHD PRS was not supported (dysphoric *v.* high symptom: *b* = 0.25, s.e. = 0.17, *t* = 1.51, *p* = 0.13; threat reactivity *v.* high symptom: *b* = 0.09, s.e. = 0.18, *t* = 0.50, *p* = 0.62); nor was the prediction that the dysphoric profile would have lower schizophrenia PRS than the other profiles (threat reactivity *v.* dysphoric: *b* = −0.04, s.e. = 0.15, *t* = −0.27, *p* = 0.79; high symptom *v.* dysphoric: *b* = 0.02, s.e. = 0.17, *t* = 0.11, *p* = 0.91). Sensitivity analyses confirmed that at a range of *p_t_* = 0.001–0.10, neither the ADHD nor the schizophrenia PRS differed significantly by PTSD profile.

## Discussion

This study of recently deployed soldiers provides evidence of three PTSD subtypes marked by different patterns of symptom endorsement, genetic and environmental risk factors, comorbidity, and course. The three profiles closely resemble those found in general population (Campbell *et al*., 2020; Pietrzak *et al*., 2014), first responder (Horn *et al*., 2016), and veteran samples (Byrne *et al*., 2019), strengthening the evidence for these specific manifestations of PTSD. The current analysis, based on longitudinal data, offers stronger evidence than previous cross-sectional investigations that particular types of stress exposures increase risk of specific PTSD presentations, and that certain PTSD subtypes involve more persistent symptoms. The finding of a significant cross-profile difference in polygenic risk for MDD offers preliminary validation of the proposed PTSD subtypes from a biological perspective.

Our results build on evidence (Byrne *et al*., 2019; Campbell *et al*., 2020; Horn *et al*., 2016; Pietrzak *et al*., 2014) that one common PTSD presentation consists of prominent intrusions, hyperarousal, and avoidance without a mood disturbance involving pervasive negative emotions/beliefs and anhedonia. Higher overall combat exposure, as well as exposure to specific life-threatening experiences (e.g. ‘close call’ such as having equipment shot off), was associated with membership in this threat-reactivity profile. These results converge with findings that individuals who identified their worst trauma as military-related (Byrne *et al*., 2019; Pietrzak *et al*., 2014) or whose trauma involved threat to life or witnessing death/destruction (Horn *et al*., 2016) were more likely to exhibit a threat-reactivity than a dysphoric presentation. Despite high combat exposure, members of the threat-reactivity profile had low comorbidity. They were also less likely to have had PTSD before deployment, suggesting that new-onset PTSD was disproportionately represented in the threat-reactivity profile.

Another PTSD presentation was marked by anhedonia, negative affect, irritable behavior, sleep problems, and difficulty concentrating. Members of this dysphoric profile had higher polygenic risk for MDD than members of other PTSD profiles, and some evidence suggested that they had more family history of depression than members of the threat-reactivity profile. These findings may indicate that expression of PTSD in individuals at higher genetic risk for depression takes a distinct form characterized by a broader affective disturbance and less re-experiencing of the trauma. A close relationship between the dysphoric PTSD profile and depression was also suggested by comorbidity analyses. MDD was the most common co-occurring disorder for members of this profile, with the majority meeting criteria for MDD, and one in five reporting suicidal ideation. Individuals with the dysphoric presentation also endorsed higher levels of personal life stress during deployment than members of the threat-reactivity profile, converging with evidence suggesting that this PTSD presentation is common among individuals with substantial life stress or diminished interpersonal resources (Horn *et al*., 2016). We did not find evidence to support our prediction that soldiers reporting more deaths of unit members would exhibit the dysphoric presentation. The potential link between traumatic loss and the dysphoric profile (Byrne *et al*., 2019; Pietrzak *et al*., 2014) may not be apparent among recently-deployed soldiers because loss of unit members is commingled with other trauma (e.g. threats to one's own life).

Re-experiencing symptoms, which have been conceptualized as pathognomonic of PTSD (Bar-Haim *et al*., 2021), were not especially pronounced in the dysphoric profile. Instead, many of this profile's most prominent symptoms align with diagnostic criteria or associated features of depressive disorders. Given the symptom presentation, the heightened polygenic risk for MDD, and the substantial MDD comorbidity of the dysphoric profile, it is worth investigating whether it is more aptly conceptualized and treated as a depressive disorder. Another intriguing possibility is that the dysphoric profile (and/or the high-symptom profile described below) reflects a proposed PTSD–MDD ‘biotype’ (Neria, 2021), which has been linked to distinctive alterations in resting-state functional connectivity of brain regions implicated in reward processing and fear processing. Investigation of potential differences in the neural substrates of the threat reactivity, dysphoric, and high-symptom PTSD profiles may yield further insights into the biological bases of PTSD heterogeneity.

Characteristics of the dysphoric profile may also suggest directions for inquiry into impacts of recent changes to the definition of PTSD. On one hand, the increased emphasis on negative alterations in cognition and mood in DSM-5 may bring more individuals with a dysphoric presentation into the PTSD category; whereas on the other, the requirement of at least two such symptoms could exclude some individuals with a threat-reactivity presentation from the diagnosis. In this study, members of the threat-reactivity profile exhibited marked intrusions and hyperarousal, but a minority endorsed each of the negative cognition and mood symptoms. Further inquiry should address whether the DSM-5 PTSD definition is optimized to capture key posttraumatic stress symptoms, while not requiring such a broad range of reactions that individuals with the cardinal symptoms of PTSD (e.g. re-experiencing; Bar-Haim *et al*., 2021) are excluded from the diagnostic category, or boundaries with other mental disorders are difficult to discern.

The third and rarest profile, still representing almost one-quarter of the sample, was comprised of soldiers who endorsed all PTSD symptoms at high levels. Relative to members of other profiles, members of the high-symptom profile were more likely to have experienced PTSD prior to the index deployment. Their PTSD symptoms also appeared more persistent, with 70% continuing to meet the PCL-5 criterion for probable PTSD at nine months postdeployment. Consistent with previous findings (Byrne *et al*., 2019; Horn *et al*., 2016; Pietrzak *et al*., 2014), members of the high-symptom profile had extremely high rates of comorbid MDD and GAD. They also had elevated rates of SUD and suicidal ideation, with roughly one-third meeting the criteria for SUD and one-quarter reporting suicidal thoughts. Given their MDD comorbidity, it is perhaps surprising that the high-symptom profile did not display elevated MDD PRS relative to the other profiles. We hypothesize that factors other than the polygenic risk for MDD may have disproportionately affected and contributed to MDD in this group (e.g. MDD emerging in reaction to severe/chronic PTSD or other comorbidities like SUD).

Elaboration of precision treatment approaches for PTSD is a priority, and future studies should evaluate whether PTSD typology moderates treatment response. For example, patients with a threat-reactivity presentation may have a more robust response to treatments targeting conditioned responses to trauma cues (e.g. prolonged exposure) than those with a dysphoric presentation. In contrast, patients with a dysphoric presentation may respond better to treatments focused on normalizing mood (e.g. antidepressant pharmacotherapy, behavioral activation). Patients with a high-symptom presentation require a thorough assessment of lifetime trauma and co-occurring disorders to fully contextualize their posttraumatic stress symptoms, and may be candidates for more complex treatment approaches give their extensive comorbidity. Assessing suicide risk is essential for all patients with PTSD; the high rates of suicidal ideation in the high-symptom (28%) and dysphoric (20%) profiles underscore this need.

Findings did not support hypotheses regarding polygenic risk for ADHD and schizophrenia. All profiles exhibited high levels of attentional problems, partly undermining the rationale for the prediction regarding ADHD PRS. This study complements growing research on PRSs that seeks to understand heterogeneity in other disorders (Dickinson *et al*., 2020; Dwyer *et al*., 2020). Further research is needed to replicate the MDD PRS finding and evaluate contributions of other PRSs to phenotypic diversity within PTSD. Cross-profile differences in MDD and other PRSs must also be examined among individuals of non-European ancestry.

Study limitations include reliance on self-report measures of symptoms, stressors, and parent history of depression, which are susceptible to inaccurate recall and response bias. The analysis sample was comprised of soldiers with self-reported symptoms indicating ‘probable PTSD’ per PCL-5 scoring guidelines. These soldiers had not been formally diagnosed with PTSD and it is possible that some subthreshold PTSD cases were included. Future studies of PTSD typologies should incorporate measures from other modalities (e.g. clinician-rated scales). Findings may not generalize to soldiers from socio-demographic groups that were not well-represented in the participating BCTs. Our ability to detect sex and race differences in profile membership may have been hindered by the low representation of female and Black soldiers. Moreover, only PRSs derived from individuals of European ancestry were available from public data sets. Given that PTSD symptoms were measured approximately three months after return from deployment, the study findings may not generalize to soldiers who experience a delayed onset of PTSD. Additionally, our analysis of PTSD persistence was impacted by attrition, and younger/unmarried soldiers and first-time deployers were underrepresented in the subsample used for that analysis. Finally, we did not examine possible modifying effects of other variables (e.g. treatment, subsequent traumas) on the relationship between profile membership at T2 and PTSD status at T3.

In conclusion, we found evidence of three PTSD subtypes among recently deployed soldiers that were defined by prominent intrusions, avoidance, and hyperarousal (threat reactivity), anhedonia and negative affect (dysphoric), and high levels of all PTSD symptoms (high symptom). The threat-reactivity profile was associated with high levels of combat exposure, whereas the dysphoric profile was associated with elevated polygenic risk for MDD and personal life stress. The high-symptom subtype appeared more persistent and was marked by extensive comorbidity. Overall, results suggest that phenotypic variation in PTSD is explained by a combination of genetic and environmental factors and may result in divergent patterns of illness. Further research is needed to clarify distinctions between the dysphoric PTSD profile and depressive disorders; such inquiry may inform discussion of whether the DSM-5 PTSD definition strikes an ideal balance between capturing key posttraumatic stress reactions and maintaining adequate definitional boundaries with other disorders. Additionally, investigation of the effects of PTSD typology on treatment response is critically important and has the potential to advance precision treatment efforts and thereby improve outcomes for patients with PTSD.

## Supplementary material

For supplementary material accompanying this paper visit https://doi.org/10.1017/S0033291721000428. click here to view supplementary material
