# Supplementary material for: Dissecting the heterogeneity of posttraumatic stress disorder: differences in polygenic risk, stress exposures, and course of PTSD subtypes
Source: Psychol Med. 2021 May 5;52(15):3646–54. doi: 10.1017/S0033291721000428 (PMC9772910; doi:10.1017/S0033291721000428)
Supplement: Supplementary file 1 [file S0033291721000428sup001.pdf]

Supplementary Material

Supplementary Figure 1: Symptom endorsement patterns of three latent PTSD profiles (N=423)

Supplementary Table 1: Pre-deployment characteristics of members of three latent PTSD profiles

Supplementary Table 2: Endorsement of specific combat/deployment stressors by PTSD profile

Supplementary Table 3: Sensitivity analysis in a subsample of soldiers of genetically determined European ancestry (n=250) used for polygenic risk score analysis

Supplementary Figure 1

Symptom endorsement patterns of three latent PTSD profiles (N=423)

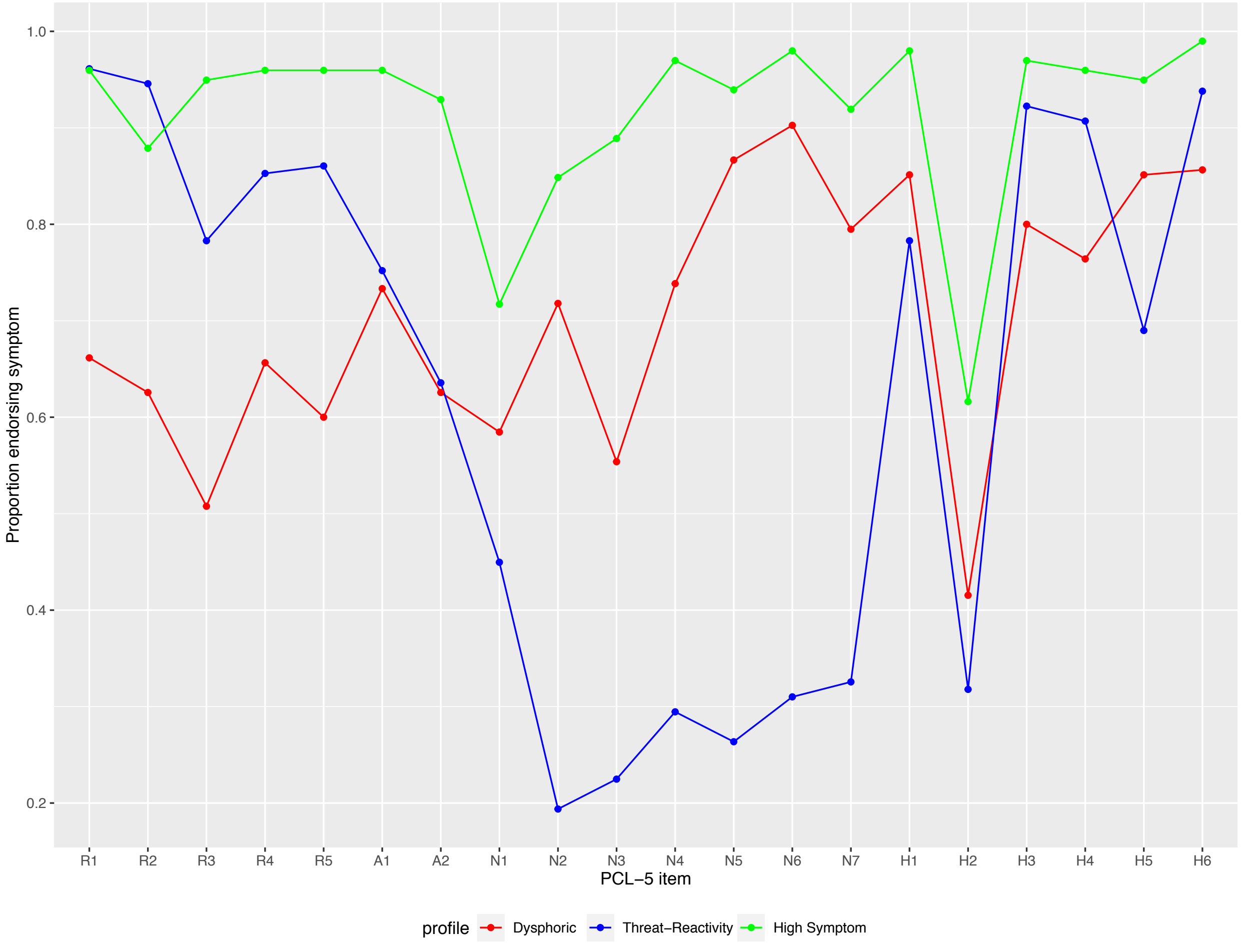

Note. Symptom endorsement is defined as a rating ≥2 ("moderately" to "extremely" bothered by the symptom) on the corresponding PCL-5 item. PTSD=posttraumatic stress disorder; PCL-5=PTSD Checklist for DSM-5; R1=intrusive memories; R2=repeated dreams; R3=flashbacks; R4=upset by reminders; R5=physical reactions to reminders; A1=avoidance of internal cues; A2= avoidance of external reminders; N1=trouble remembering the trauma; N2=strong negative beliefs; N3=blaming self or others; N4=strong negative emotions; N5=loss of interest; N6=feeling distant from others; N7=trouble experiencing positive emotions; H1=irritable behavior; H2=excessive risk-taking; H3=hypervigilance; H4= easily startled; H5=difficulty concentrating; H6=sleep problems.

Supplementary Table 1

Pre-deployment characteristics of members of three latent PTSD profiles

	Dysphoric No. (%)	Threat No. (%)	High Symptom No. (%)	*p*
Sex				0.10
Male	179 (92.3%)	125 (97.7%)	92 (92.9%)	
Female	15 (7.7%)	3 (2.3%)	7 (7.1%)	
Race				
White	142 (73.2%)	91 (70.5%)	69 (69.7%)	0.95
Black	15 (7.7%)	8 (6.2%)	7 (7.1%)	
Asian	9 (4.6%)	8 (6.2%)	7 (7.1%)	
Other	28 (14.4%)	22 (17.1%)	16 (16.2%)	
Ethnicity				
Non-Hispanic	164 (84.1%)	108 (84.4%)	80 (81.6%)	0.82
Hispanic	31 (15.9%)	20 (15.6%)	18 (18.4%)	
Age (mean, SD)	25.9 (6.2)	25.4 (5.7)	26.4 (6.1)	0.47
Educational attainment				0.80
GED	23 (11.8%)	11 (8.5%)	13 (13.3%)	
High school diploma	146 (74.9%)	101 (78.3%)	71 (72.4%)	
College degree	26 (13.3%)	17 (13.2%)	14 (14.3%)	
Marital status				0.09
Married	91 (47.4%)	74 (57.8%)	56 (57.1%)	
Never married	70 (36.5%)	43 (33.6%)	26 (26.5%)	
Previously married	31 (16.1%)	11 (8.6%)	16 (16.3%)	
Prior deployments				0.12
None	101 (52.1%)	64 (49.6%)	37 (37.4%)	
One	41 (21.1%)	34 (26.4%)	32 (32.3%)	
Two or more	52 (26.8%)	31 (24.0%)	30 (30.3%)	

Note. PTSD=posttraumatic stress disorder. Subgroup n's do not total to 423 as a result of small amounts of missing data (n=2 missing sex, n=1 missing race, n=2 missing ethnicity, n=3 missing age, n=1 missing education, n=5 missing marital status, n=1 missing number of prior deployments).

Supplementary Table 2

Endorsement of specific combat/deployment stressors by PTSD profile

	Dysphoric No. (%)	Threat No. (%)	High Symptom No. (%)	p	Pairwise results
Went on combat patrols (10+ times)				.002	**D vs. T p<.001**
No	67 (36.2%)	22 (17.9%)	27 (29.7%)		D vs. H p=.34
Yes	118 (63.8%)	101 (82.1%)	64 (70.3%)		T vs. H p=.05
Fired rounds or took enemy fire (10+ times)				.02	**D vs. T p=.010**
No	111 (60.3%)	55 (44.7%)	54 (58.7%)		D vs. H p=.80
Yes	73 (39.7%)	68 (55.3%)	38 (41.3%)		T vs. H p=.05
Was wounded (1+ times)				.14	N/A
No	159 (87.4%)	97 (78.9%)	75 (83.3%)		
Yes	23 (12.6%)	26 (21.1%)	15 (16.7%)		
Had a close call (e.g., equipment shot off; 2+ times)				.02	**D vs. T p=.014**
No	116 (62.7%)	60 (48.4%)	59 (64.1%)		D vs. H p=.90
Yes	69 (37.3%)	64 (51.6%)	33 (35.9%)		T vs. H p=.03
Unit members seriously wounded/killed (5+ times)				.02	D vs. T p=.04
No	171 (92.4%)	105 (84.7%)	88 (95.7%)		D vs. H p=.44
Yes	14 (7.6%)	19 (15.3%)	4 (4.3%)		**T vs. H p=.01**
Responsible for death of enemy combatant (1+ times)				.09	N/A
No	121 (65.8%)	66 (53.7%)	58 (63.0%)		
Yes	63 (34.2%)	57 (46.3%)	34 (37.0%)		
Witnessed homes/villages destroyed (1+ times)				.49	N/A
No	51 (27.7%)	27 (21.8%)	25 (27.2%)		
Yes	133 (72.3%)	97 (78.2%)	67 (72.8%)		
Saw severely wounded or dying/dead people (1+ times)				.008	**D vs. T p=.003**
No	155 (83.8%)	86 (69.4%)	68 (73.9%)		D vs. H p=.06
Yes	30 (16.2%)	38 (30.6%)	24 (26.1%)		T vs. H p=.54
Was bullied or hazed (1+ times)				.16	N/A
No	140 (75.7%)	105 (84.7%)	72 (78.3%)		
Yes	45 (24.3%)	19 (15.3%)	20 (21.7%)		

Note. Item-level scoring is described in a prior report (Campbell-Sills et al., 2018). Some stressors assessed in the T1 survey (sexual assault, physical assault, responsibility for the death of a noncombatant, and responsibility for the death of an ally) were too rarely reported to analyze. Bold type denotes pairwise comparisons that were significant after Bonferroni correction ($p<.017$). The n's do not total to 423 because between 22 and 28 participants were missing data on each item. PTSD=posttraumatic stress disorder; D=dysphoric profile; T=threat profile; H=high symptom profile.

Supplementary Table 3

Sensitivity analysis in a subsample of soldiers of genetically determined European ancestry (n=250) used for polygenic risk score analysis

	Dysphoric No. (%)	Threat No. (%)	High Symptom No. (%)	*p*	Pairwise results
Sex				.86	N/A
Male	114 (94.2%)	71 (95.9%)	51 (96.2%)		
Female	7 (5.8%)	3 (4.1%)	2 (3.8%)		
Age (mean, SD)	26.1 (6.3)	24.7 (5.0)	25.9 (5.7)	.24	N/A
Educational attainment				.34	N/A
GED	17 (13.9%)	6 (8.0%)	3 (5.7%)		
High school diploma	88 (72.1%)	62 (82.7%)	42 (79.2%)		
College degree	17 (13.9%)	7 (9.3%)	8 (15.1%)		
Marital status				.12	N/A
Married	54 (45.4%)	42 (56.0%)	30 (56.6%)		
Never married	43 (36.1%)	27 (36.0%)	13 (24.5%)		
Previously married	22 (18.5%)	6 (8.0%)	10 (18.9%)		
Prior deployments				.12	N/A
None	64 (52.9%)	34 (45.3%)	17 (32.1%)		
One	27 (22.3%)	22 (29.3%)	20 (37.7%)		
Two or more	30 (24.8%)	19 (25.3%)	16 (30.2%)		
Pre-deployment lifetime PTSD at T0				.002	D vs. T p=.084
No	78 (63.9%)	57 (76.0%)	24 (45.3%)		D vs. H p=.030
Yes	44 (36.1%)	18 (24.0%)	29 (54.7%)		**T vs. H p<.001**
Parental history of depression at T0				.15	N/A
No	82 (67.2%)	60 (80.0%)	37 (69.8%)		
Yes	40 (32.8%)	15 (20.0%)	16 (30.2%)		
Combat exposure at T1 (mean, SD)	3.9 (2.3)	5.0 (2.1)	4.0 (2.1)	.003	**D vs. T p<.001**<br>D vs. H p=.84<br>**T vs. H p=.009**
Personal life stress at T1 (median, IQR)	5.0 (2.0-8.0)	3.0 (2.0-6.0)	5.0 (2.5-9.0)	.085	**N/A**
Went on combat patrols (10+ times)				.031	**D vs. T p=.013**
No	34 (30.1%)	10 (13.7%)	11 (23.4%)		D vs. H p=.44
Yes	79 (69.9%)	63 (86.3%)	36 (76.6%)		T vs. H p=.22

Fired rounds or took enemy fire (10+ times)				.13	N/A
No	59 (52.7%)	28 (38.9%)	26 (54.2%)		
Yes	53 (47.3%)	44 (61.1%)	22 (45.8%)		
Was wounded (1+ times)				.19	N/A
No	93 (83.8%)	56 (76.7%)	43 (89.6%)		
Yes	18 (16.2%)	17 (23.3%)	5 (10.4%)		
Had a close call (2+ times)				.033	D vs. T p=.071
No	66 (58.4%)	32 (43.8%)	32 (66.7%)		D vs. H p=.38
Yes	47 (41.6%)	41 (56.2%)	16 (33.3%)		**T vs. H p=.016**
Unit members seriously wounded/killed (5+ times)				.034	D vs. T p=.079
No	102 (90.3%)	59 (80.8%)	46 (95.8%)		D vs. H p=.35
Yes	11 (9.7%)	14 (19.2%)	2 (4.2%)		T vs. H p=.026
Responsible for death of enemy combatant (1+ times)				.27	N/A
No	65 (58.0%)	33 (45.8%)	25 (52.1%)		
Yes	47 (42.0%)	39 (54.2%)	23 (47.9%)		
Witnessed homes/villages destroyed (1+ times)				.82	N/A
No	27 (24.1%)	15 (20.5%)	12 (25.0%)		
Yes	85 (75.9%)	58 (79.5%)	36 (75.0%)		
Saw severely wounded/dying/dead people (1+ times)				.073	**N/A**
No	91 (80.5%)	48 (65.8%)	35 (72.9%)		
Yes	22 (19.5%)	25 (34.2%)	13 (27.1%)		
Was bullied or hazed (1+ times)				.82	N/A
No	87 (77.0%)	59 (80.8%)	38 (79.2%)		
Yes	26 (23.0%)	14 (19.2%)	10 (20.8%)		
PCL-5 total score (mean, SD) at T2	38.9 (5.5)	38.8 (6.6)	62.1 (7.8)	<.001	D vs. T p=.95<br>**D vs. H p<.001**<br>**T vs. H p<.001**
Standardized PCL-5 intrusions score (mean, SD) at T2	-0.70 (0.65)	0.34 (0.78)	0.95 (0.89)	<.001	**D vs. T p<.001**<br>**D vs. H p<.001**<br>**T vs. H p<.001**
Standardized PCL-5 avoidance score (mean, SD) at T2	-0.43 (0.85)	-0.07 (1.1)	1.00 (0.69)	<.001	D vs. T p=.018<br>**D vs. H p<.001**<br>**T vs. H p<.001**
Standardized PCL-5 negative affect score (mean, SD) at T2	-0.11 (0.64)	-0.80 (0.64)	1.22 (0.79)	<.001	**D vs. T p<.001**<br>**D vs. H p<.001**

					**T vs. H p<.001**
Standardized PCL-5 anhedonia score (mean, SD) at T2	0.21 (0.67)	-1.06 (0.64)	1.02 (0.74)	<.001	**D vs. T p<.001**<br>**D vs. H p<.001**<br>**T vs. H p<.001**
Standardized PCL-5 externalizing behavior score (mean, SD) at T2	-0.18 (0.82)	-0.26 (1.07)	0.86 (0.94)	<.001	D vs. T p=.59<br>**D vs. H p<.001**<br>**T vs. H p<.001**
Standardized PCL-5 anxious arousal score (mean, SD) at T2	-0.44 (0.93)	0.23 (0.93)	0.64 (0.94)	<.001	**D vs. T p<.001**<br>**D vs. H p<.001**<br>**T vs. H p=.016**
Standardized PCL-5 dysphoric arousal score (mean, SD) at T2	-0.13 (0.94)	-0.04 (0.90)	0.83 (0.74)	<.001	D vs. T p=.48<br>**D vs. H p<.001**<br>**T vs. H p<.001**
Comorbid major depressive disorder at T2				<.001	**D vs. T p<.001**<br>**D vs. H p=.001**<br>**T vs. H p<.001**
No	49 (40.2%)	56 (74.7%)	8 (15.1%)		
Yes	73 (59.8%)	19 (25.3%)	45 (84.9%)		
Comorbid generalized anxiety disorder at T2				<.001	**D vs. T p=.004**<br>**D vs. H p<.001**<br>**T vs. H p<.001**
No	69 (56.6%)	58 (77.3%)	10 (18.9%)		
Yes	53 (43.4%)	17 (22.7%)	43 (81.1%)		
Comorbid substance use disorder at T2				.003	D vs. T p=.080<br>D vs. H p=.038<br>**T vs. H p<.001**
No	97 (79.5%)	67 (89.3%)	34 (64.2%)		
Yes	25 (20.5%)	8 (10.7%)	19 (35.8%)		
Comorbid suicidal ideation at T2				<.001	**D vs. T p<.001**<br>D vs. H p=.34<br>**T vs. H p<.001**
No	96 (78.7%)	72 (96.0%)	38 (71.7%)		
Yes	26 (21.3%)	3 (4.0%)	15 (28.3%)		
Probable PTSD at T3				.001	D vs. T p=.057<br>**D vs. H p<.001**<br>T vs. H p=.135
No	51 (60.7%)	24 (42.9%)	11 (26.8%)		
Yes	33 (39.3%)	32 (57.1%)	30 (73.2%)		

Note. Bold type denotes pairwise comparisons that were significant after Bonferroni correction (p<.017). Due to missing data, n's for some variables do not total to 250. PTSD=posttraumatic stress disorder; D=dysphoric profile; T=threat profile; H=high symptom profile.
